# Supplementary material for: Resolving the full spectrum of human genome variation using Linked-Reads
Source: Genome Res. 2019 Apr;29(4):635–45. doi: 10.1101/gr.234443.118 (PMC6442396; doi:10.1101/gr.234443.118)
Supplement: Supplemental Material [file supp_gr.234443.118_Supplemental_longranger-2.2.zip › longranger-2.2.tar.bz2!/lr2.2-public/lib/stan/cmdstan/stan/doc/api/html/autocorrelation_8hpp_source.html]

Stan: src/stan/prob/autocorrelation.hpp Source File


|  |  |
| --- | --- |
|  | Stan  2.5.0  probability, sampling & optimization |


- Main Page
- Related Pages
- Namespaces
- Classes
- Files

- File List
- File Members

All Classes Namespaces Files Functions Variables Typedefs Enumerations Enumerator Friends Macros Pages

- src
- stan
- prob

autocorrelation.hpp

Go to the documentation of this file.

1 #ifndef STAN\_\_PROB\_\_AUTOCORRELATION\_HPP

2 #define STAN\_\_PROB\_\_AUTOCORRELATION\_HPP

3

4 #include <stan/math/matrix.hpp>

5 #include <stan/math/matrix/mean.hpp>

6

7 #include <vector>

8 #include <complex>

9 #include <unsupported/Eigen/FFT>

10

11

12 namespace stan {

13

14  namespace prob {

15

16  namespace {

21  size\_t fft\_next\_good\_size(size\_t N) {

22  if (N <= 2) return 2;

23  while (true) {

24  size\_t m = N;

25  while((m % 2) == 0) m /= 2;

26  while((m % 3) == 0) m /= 3;

27  while((m % 5) == 0) m /= 5;

28  if (m <= 1)

29  return N;

30  N++;

31  }

32  }

33  }

34

55  template <typename T>

56  void autocorrelation(const std::vector<T>& y,

57  std::vector<T>& ac,

58  Eigen::FFT<T>& fft) {

59

60  using std::vector;

61  using std::complex;

62

63  size\_t N = y.size();

64  size\_t M = fft\_next\_good\_size(N);

65  size\_t Mt2 = 2 \* M;

66

67

68  vector<complex<T> > freqvec;

69

70  // centered\_signal = y-mean(y) followed by N zeroes

71  vector<T> centered\_signal(y);

72  centered\_signal.insert(centered\_signal.end(),Mt2-N,0.0);

73  T mean = stan::math::mean(y);

74  for (size\_t i = 0; i < N; i++)

75  centered\_signal[i] -= mean;

76

77  fft.fwd(freqvec,centered\_signal);

78  for (size\_t i = 0; i < Mt2; ++i)

79  freqvec[i] = complex<T>(norm(freqvec[i]), 0.0);

80

81  fft.inv(ac,freqvec);

82  ac.resize(N);

83

84  /\*

85  vector<T> mask\_correction\_factors;

86  vector<T> mask;

87  mask.insert(mask.end(),N,1.0);

88  mask.insert(mask.end(),N,0.0);

89

90  freqvec.resize(0);

91  fft.fwd(freqvec,mask);

92  for (size\_t i = 0; i < Nt2; ++i)

93  freqvec[i] = complex<T>(norm(freqvec[i]), 0.0);

94

95  fft.inv(mask\_correction\_factors, freqvec);

96

97  for (size\_t i = 0; i < N; ++i) {

98  ac[i] /= mask\_correction\_factors[i];

99  }

100  \*/

101  for (size\_t i = 0; i < N; ++i) {

102  ac[i] /= (N - i);

103  }

104  T var = ac[0];

105  for (size\_t i = 0; i < N; ++i)

106  ac[i] /= var;

107  }

108

125  template <typename T>

126  void autocorrelation(const std::vector<T>& y,

127  std::vector<T>& ac) {

128  Eigen::FFT<T> fft;

129  return autocorrelation(y,ac,fft);

130  }

131

132

133  }

134 }

135

136 #endif

mean.hpp

stan::math::mean

boost::math::tools::promote\_args< T >::type mean(const std::vector< T > &v)

Returns the sample mean (i.e., average) of the coefficients in the specified standard vector...

**Definition:** mean.hpp:23

stan::prob::autocorrelation

void autocorrelation(const std::vector< T > &y, std::vector< T > &ac, Eigen::FFT< T > &fft)

Write autocorrelation estimates for every lag for the specified input sequence into the specified res...

**Definition:** autocorrelation.hpp:56

matrix.hpp


---

[ Stan Home Page ]

*© 2011–2014,
Stan Development Team.*
